# Satisfaction and Acceptability Ratings of a Web-Based Self-help Intervention for Depression: Retrospective Cross-sectional Study From a Resource-Limited Country

**DOI:** 10.2196/29566

**Published:** 2022-04-04

**Authors:** Ma. Asunción Lara, Pamela Patiño, Marcela Tiburcio, Laura Navarrete

**Affiliations:** 1 Department of Psychosocial Studies in Specific Population Division of Epidemiological and Psychosocial Research Ramón de la Fuente Muñiz National Institute of Psychiatry Mexico City Mexico; 2 Division of Epidemiological and Psychosocial Research Ramón de la Fuente Muñiz National Institute of Psychiatry Mexico City Mexico; 3 Department of Social Sciences in Health Division of Epidemiological and Psychosocial Research Ramón de la Fuente Muñiz National Institute of Psychiatry Mexico City Mexico

**Keywords:** depression, web-based intervention, unguided intervention, acceptability, satisfaction, resource-limited country

## Abstract

**Background:**

Web-based interventions are at an early stage in non–English-speaking low- and middle-income countries, where they remain scarce. Help for Depression (HDep) is one of the few unguided web-based interventions available in Latin America. The results of a use/usability analysis of the original version served as the basis for generating a more user-friendly second version.

**Objective:**

The aim of this study is to explore participants’ satisfaction and acceptability for the second version of HDep.

**Methods:**

A retrospective cross-sectional design was used. An email invitation to complete a web-based survey was sent to all people who accessed HDep in 2018. The questionnaire included satisfaction and acceptability scales and open-ended questions. Complete questionnaires were retrieved from 191 participants: 35.1% (67/191) from those who visited only the home page (home page users [HPUs]) and 6.47% (124/1916) from those who registered to use the program (program users [PUs]).

**Results:**

In all groups, users experienced high levels of depressive symptoms (189/191, 98.9%; Center for Epidemiological Studies Scale-Depression >16). Moderate levels of satisfaction (HPUs: mean 21.9, SD 6.7; PUs: mean 21.1, SD 5.8; range: 8-32) and acceptability (HPUs: mean 13.8, SD 3.9; PUs: mean 13.9, SD 3.2; range: 5-20) were found in both groups. Logistic regression analyses showed that among HPUs, women were more satisfied with HDep (odds ratio [OR] 3.4, 95% CI 1.1-10.0), whereas among PUs, older respondents (OR 1.04, 95% CI 1.01-1.08), those with paid work (OR 3.1, 95% CI 2.4-7.6), those who had not been in therapy (OR 2.42, 95% CI 1.09-5.98), and those who had not attempted suicide (OR 3.4, 95% CI 1.1-11.1) showed higher satisfaction. None of the sociodemographic/mental health variables distinguished the acceptability ratings among HPUs. Among PUs, those with paid work (OR 2.5, 95% CI 1.1-5.5), those who had not been in therapy (OR 3.1, 95% CI 1.3-7.3), those without disability (OR 2.9, 95% CI 1.3-6.6), and those who had not attempted suicide (OR 2.6, 95% CI 1.0-6.6) showed higher acceptability.

**Conclusions:**

HDep has good levels of satisfaction and acceptability for approximately half of its users, and the information provided by respondents suggested feasible ways to remedy some of the deficiencies. This qualitative–quantitative study from a low- to middle-income, non–English-speaking country adds to existing knowledge regarding acceptance and satisfaction with web-based interventions for depression in resource-limited countries. This information is important for the creation and adaptation of web-based interventions in low- and middle-income countries, where access to treatment is a major concern, and web-based prevention and treatment programs can help deliver evidence-based alternatives. It is necessary to document the pitfalls, strengths, and challenges of such interventions in this context. Understanding how users perceive an intervention might suggest modifications to increase adherence.

## Introduction

### Background

The World Health Organization [1] has ranked depression as the single largest contributor to global disability, accounting for 7.5% of all years lived with disability. More than 80% of this nonfatal disease burden occurs in low- and middle-income countries (LMICs). In Mexico, depressive disorders account for 8.6% of the years lived with disability [1]. Prevention and treatment interventions can reduce the burden associated with depression and other mental health disorders. However, mental disorders remain untreated in many nations; in Latin America, only 5% of people with affective disorders receive adequate treatment [2]. In Mexico, only 6.4% of those diagnosed with major depressive disorder receive minimally adequate treatment [3].

Internet- or web-based interventions represent effective, accessible, and low-cost means to treat and prevent depressive disorders, and they can be broadly disseminated [4,5]. Unguided interventions, based purely on self-help with no human support, can reach large numbers of people at low cost, and there is evidence that even those who experience enough symptoms to screen positive for a major depressive episode use preventive interventions [5]. Meta-analytic studies show that these interventions have benefits but exhibit low adherence rates [6]. The average rate of adherence in unguided interventions is estimated at 26%, compared with 72% in guided interventions [7]. Despite the high attrition rates and lesser effectiveness of unguided programs, these low-cost, low-intensity, web-based interventions are suitable from a public health perspective as early intervention in a stepped-care process [8] and are thus of particular benefit in LMICs [9].

Web-based interventions for common mental health problems, such as depression, have a long history and have evolved rapidly in high-income countries, but they are at an earlier stage of development and are less common in LMICs [10-12]. Web-based interventions to prevent depression are associated with small but positive effects on the symptoms [13]. Help for Depression (HDep; Ayuda para Depresión) [14], in Mexico, was the first such web-based intervention in Latin America [15].

The initial version of HDep (2009-2013) was modeled after a face-to-face psychoeducational intervention in Mexico to prevent depression in women, based on multimodal and cognitive behavioral principles (Table 1) [16]. This initial intervention was modeled after that of Muñoz and Ying [17] in California, designed for the ethnic minority groups, including the Latino population. The content of the program that we developed went through a step-by-step process including focus groups and open-ended questionnaires to verify that language, illustrations, and content were sensitive to the target population [18].

**Table 1:** Help for Depression content.

Module	Content
1. What is depression?	Diagnosis Symptoms Risk factors
2. Identify negative thoughts	Relationship between ways of thinking and depression Identifying your ways of thinking
3. How to transform negative thoughts	Changing negative thought patterns Questioning your negative thoughts Transforming your negative thoughts
4. Childhood experiences	Your thought patterns derived from your childhood experiences Transforming your negative thoughts Reinforcing your positive thoughts in daily life
5. Adverse events	Adverse life events Stressful events in everyday life Adverse events and negative thoughts Relaxation exercise
6. Other strategies to improve mood	Increasing social support Behavioral activation

**Figure 1:**
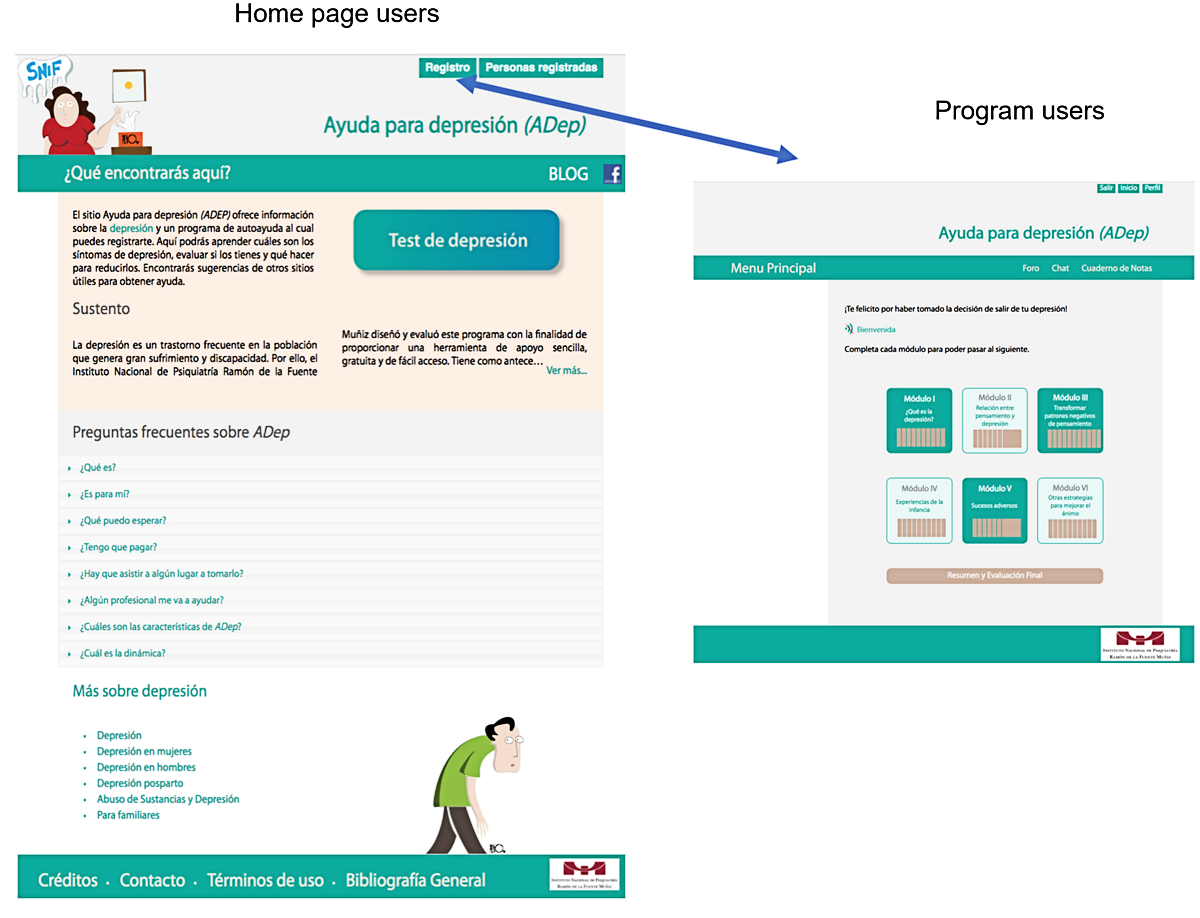
Help for depression home page: types of users of the intervention.

A use/usability analysis of the first version of HDep showed that 15.6% of the users were men [15]. Two psychologists with experience working with men reviewed the language and vignettes for cultural sensitivity to male users. The analysis of that version also suggested the need for a shorter and more user-friendly version [15], which was produced by reducing the number of modules. In the new version, users need to provide minimal information (sex, age, and email) to access home page content. The home page includes (1) a self-assessment scale for depressive symptoms (Center for Epidemiological Studies Scale-Depression [CES-D]), with feedback provided via email; (2) extensive information on depression and places to receive help; (3) a description of the aims and content of the intervention; and (4) a link to register to use the program modules. This arrangement allows for two types of users: home page users (HPUs), who are those who prefer to visit only the home page or landing page and program users (PUs), who decide to register for the program modules after exploring the home page (Figure 1). PUs are asked to provide additional information (marital status, education, psychological or psychiatric treatment for depression, suicide attempts, alcohol and drug use, and medication for mental health problems). HPUs and PUs who answer the CES-D and other health measures are given feedback on their responses; those who may be at risk for clinical depression or mental health disorders are advised to find additional psychological or psychiatric help. HDep is freely accessible, with no inclusion criteria for registration; it is thus available not only for people who meet a clinical diagnosis of depression but also to those with other mental health problems, such as substance abuse [15].

The lack of adequate mental health services for most of the population of Mexico and other Latin American countries means that very few people receive even minimally adequate treatment for depression [3]. In this context, internet-based interventions play an important role in the prevention and treatment of depression; however, they are still at an early stage of development in these countries.

Since 2014, the revised version of HDep has had around 2956 visits each month from individuals who answered the depression scale (CES-D) and received feedback based on their scores. At the same time, there is a high rate of attrition, which is consistent with data from a scoping review in Latin America [19]. To improve adherence, it is important to analyze users’ experience, perception, and satisfaction with HDep to find ways to encourage people to complete the intervention [5].

### Objectives

The aim of this study is to assess the acceptability of and satisfaction with the updated version of HDep, considering both types of users separately: HPUs and PUs. This was an exploratory study, with the underlying hypothesis that PUs would show greater acceptance and satisfaction than HPUs, as they were receiving a higher dose of the intervention.

## Methods

### Study Design and Participants

**Figure 2:**
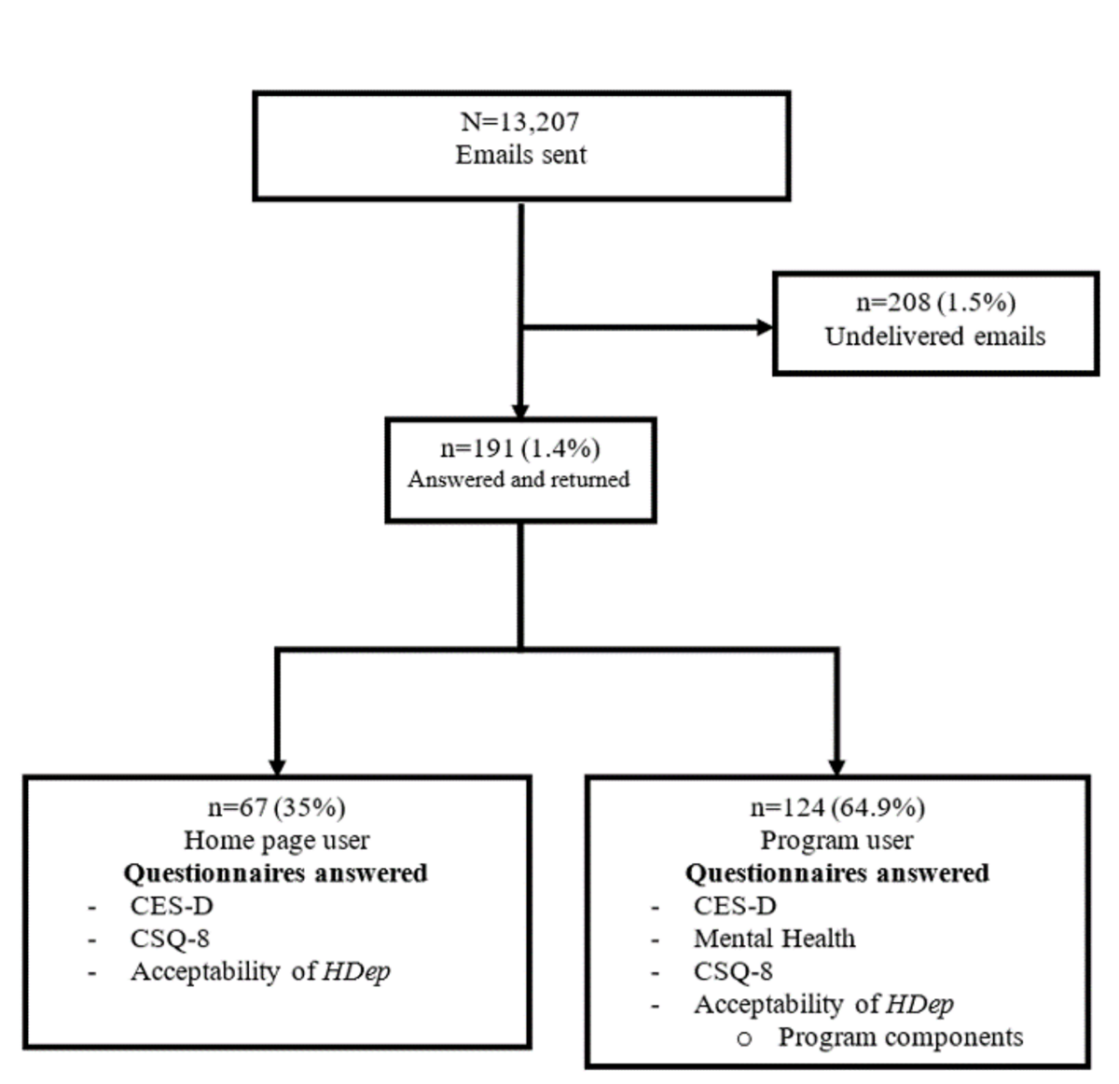
Participant flowchart. CES-D: Center for Epidemiological Studies Scale-Depression; CSQ-8: Client Satisfaction Questionnaire-8; HDep: Help for Depression.

A retrospective cross-sectional design was used in this study. The population corresponds to people who visited HDep: HPUs and PUs from January to December 2018 (N=13,207) and answered the initial depressive symptoms questionnaire (CES-D), which is accessible on the home page. The sample size was determined according to the procedure of Lemeshow et al [20] for the following parameters: a finite population with a CI of 95% and a margin of error of 10%. This margin of error was chosen because web surveys have response rates approximately 12% points lower than other survey modes [21]. The minimum number of responses required was 96. Of the 13,207 emailed questionnaires, 191 (191/13,207, 1.45%) were answered and returned. A total of 208 (208/13,207, 1.57%) were undelivered (indicated by automatic replies). There is no information on whether the rest reached the target users (Figure 2). The CI for the response rate was 95%, with a margin of error of 7%.

### Instruments

Data retrieved from HDep databases: email, sex, age, marital status, occupation, education, depressive symptoms (CES-D), disability during the previous month owing to depressive symptoms, psychological or psychiatric treatment for depression, suicide attempt in the last 6 months, alcohol and drug use, and medication [15].

#### Depressive Symptoms

Depressive symptoms are assessed with the CES-D [22], which has 20 items with Likert response options (0=rarely or never to 3=most of the time). The cutoff point indicating the presence of depressive symptoms was ≥16. The CES-D has been validated in Mexico [23].

#### Satisfaction With HDep

Satisfaction with HDep is measured with the short version of the Client Satisfaction Questionnaire-8 (CSQ-8) [24] (Spanish version [25]), which includes 8 questions on a Likert scale of 1 to 4, with higher scores indicating greater satisfaction. A change was made in the wording of the questions to align it with the purpose of the study: *HDep* was used instead of *service*. The CSQ-8 is reliable across a variety of ethnic contexts, including Hispanic groups, and the Spanish version is as reliable as the English version (Cronbach *α*=.90) [26]. The Cronbach coefficient for the scale in the sample was a Cronbach *α* of .95.

#### Acceptability of HDep

As in many studies [27,28], an ad hoc questionnaire was developed to evaluate this construct. It consisted of 17 questions, of which only 15 quantitative questions were analyzed (the other two were open-ended questions that received few responses; thus, they were not included: *Is there something additional that helped you that is not listed here?* and *Are there any additional comments you would like to add?*). The first five questions were related to general acceptability: *Is the home page persuasive? Is the page layout of the modules inviting? Does HDep seem useful for managing depression? Did HDep meet your expectations?* and *Is the website user friendly?* Respondents were asked to answer the questions on a 4-point Likert scale (1=low to 4=high acceptability). The Cronbach coefficient for these 5 questions in the sample was a Cronbach *α* of .89. In all, 2 questions rated the HDep content and design on a scale of 1 to 10, with a space to provide open-ended elaboration. The final eight questions scored the following elements of HDep on a scale of 1 to 10: module information, sample cases, activities, forums, chats, thought charts, audio, and depressive symptom assessment and feedback. The Cronbach coefficient for the scale in the sample was a Cronbach *α* of .95. Except for the depressive symptoms assessment and feedback, these questions were analyzed only for users who registered for the program (PUs), as these elements are part of the program modules.

### Procedure

The link to the web survey was delivered via email (using the Mail Chimp and Google Forms platforms), including a cover letter explaining the objectives of the study and why users were being contacted. A follow-up email was sent 1 month later to those who had not answered. As a token of appreciation, those who responded to the survey were sent a list of 10 positive thoughts to reinforce HDep activities and improve mood.

The survey was designed to be user friendly; it was configured to prevent users from leaving questions unanswered.

### Ethical Considerations

The study was approved by the Ethics Committee of the National Institute of Psychiatry, Mexico (CEI/C/050/2018). The terms of use on the HDep home page explain that some of the data provided by users may be used for scientific reports and publications but that participant confidentiality will be maintained. An informed consent letter was included with the survey, underscoring the guarantee of confidentiality.

### Data Analyses

The percentages of sociodemographic characteristics and the means and SDs of the overall satisfaction scale and for individual items were obtained for HPUs and PUs and compared using chi-square and 2-tailed *t* tests. The same procedure was followed to evaluate the elements of acceptability (user friendly, scope, usefulness, motivation to register and carry out the activities, and expectations) of HDep overall and for each component. Logistic regression analyses were conducted to assess the characteristics of users who were satisfied with the HDep program among the HPUs and PUs. For these analyses, the acceptability and satisfaction scales were dichotomized: the cutoff points were defined at the 75th percentile for each scale (for the CSQ-8, it was ≥26 and for the scale of acceptability, ≥16). Finally, a qualitative thematic analysis was conducted on the responses to the 2 open-ended questions regarding design and content.

## Results

### Demographic and Psychological Characteristics

Of the 13,207 emailed invitations, 191 (1.45%) were completed questionnaires; 67 (67/191, 35.1%) were from HPUs and 124 (124/191, 64.9%) from PUs. Most were women (141/191, 73.8%; HPUs: 42/67, 63%; PUs: 99/124, 79.8%; *χ*^2^_1_=6.22; *P*=.01) and aged 20 years (57/191, 29.8%; HPUs: 24/67, 36%; PUs: 33/124, 26.6%) or aged 21-30 years (68/191, 35.6%; HPUs: 24/67, 36%; PUs: 44/124, 35.5%; *χ*^2^_1_=6.22; *P*=.01).

Nearly all users (189/191, 98.9%) experienced high levels of depressive symptoms (CES-D>16), with no significant difference between PUs (124/124, 100%) and HPUs (65/67, 97%; *χ*^2^_1_=3.74; *P*=.12). Questions on mental health problems were answered only by PUs; 70 (70/124, 56.4%) PUs reported disability associated with depressive symptoms, 20 (20/124, 16.1%) PUs reported psychological or psychiatric treatment for depression, 21 (21/124, 16.9%) PUs reported a previous suicide attempt, 27 (27/124, 21.8%) PUs reported excessive alcohol use, 15 (15/124, 12.1%) PUs reported excessive drug use, and 39 (39/124, 31.5%) PUs had received drug treatment (Table 2).

**Table 2:** Demographic and psychological characteristics.

Characteristics				All (N=191), n (%)		Home page user (n=67), n (%)		Program user (n=124), n (%)		Values		
										Chi-square (*df*)		*P* value
**Demographic characteristics**												
	**Sex**									6.22 (1)		.01
		Male	50 (26.2)		25 (37.3)		25 (20.2)					
		Female	141 (72.8)		42 (62.7)		99 (79.8)					
	**Age (years)**									3.23 (3)		.35
		20	57 (29.8)		24 (35.8)		33 (26.6)					
		21-30	68 (35.6)		24 (35.8)		44 (35.5)					
		31-40	34 (17.8)		8 (11.9)		26 (21)					
		41	32 (16.7)		11 (16.4)		21 (16.9)					
	**Marital status**									N/A^a^		N/A
		Single	N/A		N/A		87 (70.2)					
		With partner	N/A		N/A		37 (29.8)					
	**Occupation**									N/A		N/A
		Employed	N/A		N/A		57 (46)					
		Unemployed	N/A		N/A		67 (54)					
	**Education**									N/A		N/A
		Junior high school or less	N/A		N/A		10 (5.2)					
		High school or more	N/A		N/A		181 (94.8)					
**Psychological characteristics**												
	Depressive symptoms (CES-D^b^>16)		189 (98.9)		65 (97)		124 (100)		3.74 (1)		.12	
	Disability the previous month owing to depressive symptoms		N/A		N/A		70 (56.5)		N/A		N/A	
	**Psychological or psychiatric treatment for depression**									N/A		N/A
		Yes, currently	N/A		N/A		20 (16.1)					
		Not currently but in the past	N/A		N/A		32 (25.5)					
		No	N/A		N/A		72 (58.1)					
	Suicide attempt in the last 6 months		N/A		N/A		21 (16.9)		N/A		N/A	
	Alcohol use		N/A		N/A		27 (21.8)		N/A		N/A	
	Drug use		N/A		N/A		15 (12.1)		N/A		N/A	
	Medication (for feeling nervous, anxious, or excessively energetic)		N/A		N/A		39 (31.5)		N/A		N/A	

^a^N/A: not applicable.

^b^CES-D: Center for Epidemiological Studies Scale-Depression.

### Satisfaction With HDep

The mean satisfaction with HDep was 21.9 (SD 6.7; range 8-32) for HPUs and 21.1 (SD 5.8; range 8-32) for PUs. No significant difference was found in the overall level of satisfaction among types of users (*t*_189_=0.845; *P*=.39; Table 3) or on individual items. The highest scores were given to *If a friend needed help, would you recommend HDep?* and *If you were to seek help again, would you come back to HDep*? The item with the lowest score was *To what extent did HDep meet your needs?* (Table 3). No significant differences were found between HPUs and PUs on individual items.

**Table 3:** Satisfaction with Help for Depression (N=191).

Item	Home page user (n=67), mean (SD)	Program user (n=124), mean (SD)	Values	
			*t* test (*df*)	*P* value
Scale mean	21.90 (6.74)	21.10 (5.83)	0.84 (189)	.39
1. How would you rate the quality of help you have received?	2.87 (0.88)	2.73 (0.79)	1.04 (189)	.29
2. Did you get the kind of help you wanted?	2.58 (1.00)	2.51 (0.87)	0.53 (189)	.95
3. To what extent has the program helped to solve your problems?	2.21 (0.89)	2.07 (0.77)	1.05 (189)	.27
4. If a friend were in need of similar help, would you recommend our program to them?	3.04 (0.89)	3.00 (0.79)	0.35 (189)	.72
5. How satisfied are you with the amount of help you have received?	2.84 (0.97)	2.64 (0.82)	1.49 (189)	.13
6. Has the help you received helped you to deal better with your problems?	2.60 (0.90)	2.60 (0.79)	0.06 (189)	.95
7. Overall, how satisfied are you with the service you have received?	2.72 (1.05)	2.65 (0.94)	0.47 (189)	.63
8. If you needed to seek help again, would you come back to this program?	3.04 (1.03)	2.90 (0.89)	0.98 (189)	.32

### HDep Acceptability

The mean of general acceptability of HDep was 13.84 (SD 3.97; range 5-20) for HPUs and 13.97 (SD 3.29; range 5-20) for PUs, with no significant difference among types of users (*t*_189_=0.24; *P*=.80; Table 4). The item with the highest rating was *HDep, a user-friendly program*, and the item with the lowest rating was *HDep, which met my expectations*.

**Table 4:** Help for Depression (HDep) acceptability (N=191).

Item	Home page users (n=67), mean (SD)	Program users (n=124), mean (SD)	Values	
			*t* test (*df*)	*P* value
Scale mean	13.84 (3.97)	13.97 (3.29)	0.24 (189)	.80
1. Home page specifies what I can find in HDep and persuades me to register.	2.81 (0.89)	2.92 (0.78)	0.91 (189)	.36
2. Once I am registered, the layout of the module page motivates me to go into each module.	2.72 (0.91)	2.69 (0.77)	0.24 (189)	.80
3. HDep seems like a useful tool to manage depression on my own.	2.70 (0.87)	2.73 (0.88)	0.24 (189)	.80
4. Participating in HDep has met my expectations.	2.72 (0.90)	2.58 (0.85)	1.02 (189)	.30
5. HDep is a user-friendly program.	2.90 (0.97)	3.05 (0.80)	1.23 (189)	.21

### Profiles of Users’ Satisfaction and Acceptability With HDep

Logistic regression analyses showed that among HPUs, women (odds ratio [OR] 3.44, 95% CI 1.16-10.0) were more satisfied with HDep, whereas among PUs, older participants (OR 1.04, 95% CI 1.01-1.08), those with paid work (OR 3.12, 95% CI 2.40-7.69), those who had not been in therapy (OR 2.42, 95% CI 1.09-5.98), and those who had not attempted suicide (OR 3.44, 95% CI 1.08-11.11) showed higher satisfaction (Table 5). There were no significant differences in acceptability ratings among HPUs by sex, age, or the presence or absence of depressive symptoms. Among PUs, those with paid work (OR 2.50, 95% CI 1.16-5.55), those who had not been in therapy (OR 3.17, 95% CI 1.38-7.30), those without disability associated with depression (OR 2.94, 95% CI 1.35-6.66), and those who had not attempted suicide (OR 2.63, 95% CI 1.03-6.66) gave higher acceptability ratings (Table 6).

**Table 5:** Profile of home page users (HPUs) and program users (PUs) satisfied with Help for Depression.

Characteristics	CSQ-8^a^≥26			
	HPUs		PUs	
	OR^b^ (95% CI)	*P* value	OR (95% CI)	*P* value
Age	1.01 (0.97-1.05)	.46	1.04 (1.01-1.08)	.01
Female	3.44 (1.16-10.0)	.02	1.01 (0.36-2.89)	.98
Has partner	N/A^c^	N/A	1.51 (0.63-3.70)	.35
Employed	N/A	N/A	3.12 (2.40-7.69)	.01
High school or more	N/A	N/A	3.12 (0.38-2.5)	.29
Has not been in therapy	N/A	N/A	2.42 (1.09-5.98)	.04
Depressive symptoms	0.98 (0.94-1.03)	.37	0.97 (0.93-1.01)	.28
Without disability the previous month owing to depressive symptoms	N/A	N/A	1.85 (0.26-2.35)	.21
Without previous depression	N/A	N/A	2.5 (0.08-3.89)	.24
Medication (for feeling nervous, anxious, or excessively energetic)	N/A	N/A	2.00 (0.85-4.68)	.11
No suicide attempts	N/A	N/A	3.44 (1.08-11.11)	.03
Alcohol use	N/A	N/A	1.43 (0.55-3.72)	.45
Drug use	N/A	N/A	1.33 (0.19-2.89)	.68

^a^CSQ-8: Client Satisfaction Questionnaire-8.

^b^OR: odds ratio.

^c^N/A: not applicable.

**Table 6:** Profile of home page users (HPUs) and program users (PUs) who accepted Help for Depression (HDep).

Characteristics	Acceptability of HDep ≥16			
	HPUs		PUs	
	OR^a^ (95% CI)	*P* value	OR (95% CI)	*P* value
Age	1.01 (0.97-1.05)	.49	1.03 (0.99-1.07)	.63
Female	2.43 (0.90-7.14)	.09	3.13 (0.99-9.83)	.60
Has partner	N/A^b^	N/A	1.92 (0.85-4.34)	.11
Employed	N/A	N/A	2.50 (1.16-5.55)	.20
High school or more	N/A	N/A	2.08 (0.42-2.37)	.36
Has not been in therapy	N/A	N/A	3.17 (1.38-7.30)	.01
Depressive symptoms	0.97 (0.93-1.02)	.65	0.96 (0.93-1.00)	.26
Without disability the previous month owing to depressive symptoms	N/A	N/A	2.94 (1.35-6.66)	.01
Without previous depression	N/A	N/A	0.34 (0.74-1.62)	.18
Medication (for feeling nervous, anxious, or excessively energetic)	N/A	N/A	1.20 (0.54-2.67)	.65
No suicide attempts	N/A	N/A	2.63 (1.03-6.66)	.04
Alcohol use	N/A	N/A	1.25 (0.51-3.05)	.62
Drug use	N/A	N/A	2.17 (0.57- 8.33)	.26

^a^OR: odds ratio.

^b^N/A: not applicable.

### Acceptability of Content, Design, and Tools of the Program

Content was assessed similarly for HPUs and PUs (HPUs: mean 7.1, SD 2.9; PUs: mean 7.0, SD 2.5; range 0-10; *t*_189_=0.16; *P*=.87). The design was also evaluated positively, with means of 7 and 7.5 (HPUs: mean 7.5, SD 2.6; PUs: mean 7.0, SD 2.5), with no significant difference among types of users (*t*_189_=1.33; *P*=.18; Table 7).

HDep individual components, evaluated only by PUs, were scored from 5.19 (SD 3.31) to 6.88 (SD 3.14). Forums (mean 5.62, 3.24) and chats (mean 5.19, SD 3.31) had the lowest acceptability. Depressive symptom assessment and feedback, evaluated by both types of users, had the highest acceptability (PUs: mean 6.88, SD 3.14; HPUs: mean 6.73, SD 3.79), with no significant difference between the 2 user types (Table 7).

**Table 7:** Users’ evaluation of Help for Depression (HDep) content, design, and tools.

Item (rate from 0 to 10 how much each of the following has helped you to manage depression)		Home page users (n=67), mean (SD)	Program users (n=124), mean (SD)	Values			
				*t* test (*df*)		*P* value	
HDep content		7.1 (2.93)	7.00 (2.52)	0.16 (189)		.87	
HDep design		7.52 (2.60)	7.00 (2.55)	1.33 (189)		.18	
**HDep components**					N/A^a^		N/A
	Module information	N/A	6.52 (3.10)				
	Case samples	N/A	6.52 (3.01)				
	Activities (scales, thought charts, etc)	N/A	6.46 (3.08)				
	Forums	N/A	5.62 (3.24)				
	Chat	N/A	5.19 (3.31)				
	Thought exercises	N/A	6.35 (3.33)				
	Audio	N/A	6.09 (3.26)				
Depressive symptom assessment and feedback		6.73 (3.79)	6.88 (3.14)	0.27 (189)		.79	

^a^N/A: not applicable.

### Analysis of Open-ended Questions

#### HDep Content

**Figure 3:**
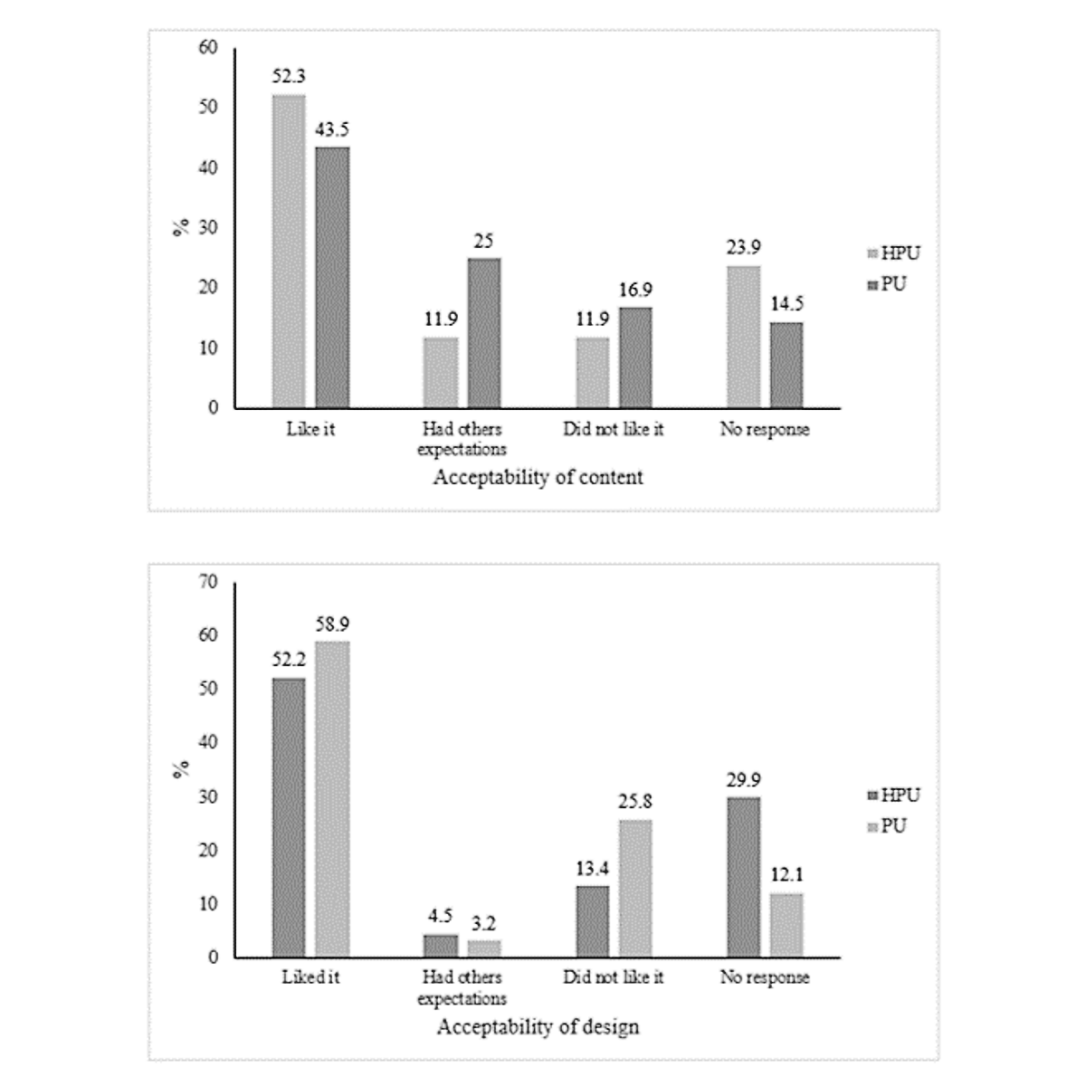
Acceptability of help for depression content and design. (A) Liked it (*χ*^2^_1_=1.32; *P*=.15); Had other expectations (*χ*^2^_1_=4.56; *P*=.02); Did not like it (*χ*^2^_1_=0.84; *P*=.24); No response (*χ*^2^_1_=2.60; *P*=.08). (B) Liked it (*χ*^2^_1_=0.77; *P*=.23); Had other expectations (*χ*^2^_1_=0.19; *P*=.47); Did not like it (*χ*^2^_1_=3.95; *P*=.03); No response (*χ*^2^_1_=9.13; *P*=.001). HPU: home page user; PU: program user.

Responses to open-ended questions fell into one of three categories: (1) Liked it, (2) Had other expectations, or (3) Did not like it. The fourth category included those who did not respond (Figure 3). Examples of these explanations are presented in Table 8.

The three categories were as follows:

1. Liked the content—approximately half of the users liked the content: 52% (35/67) of the HPUs and 43.5% (54/124) of the PUs, with no significant difference observed between the 2 groups (Figure 3). HPUs considered the content good, that the help was excellent, and that the use of cases from everyday life encouraged reflection; however, they sometimes considered the web interface cold (Table 8). PUs who liked the content found it systematic, that it gradually made it easier to face life, that the content was meaningful and friendly, and that it allowed them to measure their personal progress. Some respondents who mentioned positive features also pointed out others that required improvement, including a need for additional resources to strengthen individual commitment and motivation.
2. Had other expectations—a significantly greater number of PUs had other expectations (31/124, 25%) than HPUs (8/67, 12%; Figure 3). HPUs reported signing up in search of new information and help and someone to tell them everything was okay, which they did not find. PUs also sought not only interaction with a machine but also contact with a human being, an expert, in forums and chat, with email and mobile phone reminders (Table 8).
3. Did not like the content—respondents who did not like HDep content included 12% (8/67) of HPUs and 16.9% (21/124) of PUs (Figure 3). HPUs thought HDep was neither helpful nor harmful, that they did not need *a pat on the back*, and that *a little rough treatment might not hurt*. PUs said that the content did not motivate them to continue; in the opinion of one, “there was too much content to read which you can’t when you are depressed” (Table 8).

**Table 8:** Open-ended explanations of score given to acceptability of Help for Depression content.

Category	Home page users	Program users
Liked it	“In general, its content is good.” “It seems like a very good page, with a lot of satisfactory content.” “The help has been excellent; however, I get the impression that the interface part of the webpage sometimes seems a bit cold.” “It seems to me that something important they get right is that they show phrases and cases from everyday life and that they encourage reflection on personal actions based on those examples.”	“Because it is very systematic, although some strategy is needed to strengthen individual commitment with the help.” “When I opened the page, I doubted it would help me. But as I did the exercises I realized that, although it was very gradual, my way of facing life was getting easier. The problems were getting smaller.” “The content seemed good to me, but not so motivating.” “The content is very meaningful and concrete, the way it is presented is very friendly and allows us to keep measuring our personal results.” “I understand that my level is very high, and there is support, but I think there should be other tools to address the problem, even if the level of depression is as high as mine. It is not a bad option, it is a good one, however, I would recommend increasing or improving the strategy a bit.”
Had other expectations	“I signed up for news and help, but it never got to me.” “They didn’t give me any advice, they just gave me a number to search for psychologists in my city. I even said that all I wanted was for someone to tell me that everything was going to be okay.”	“For a topic like this, the human part is necessary, the information is good, but only being able to interact with a machine is cold, at critical times you need to be able to be heard by a person.” “Both in the forum and in the chat, there needs to be an expert moderator, since the participants share personal and often erroneous information.” “I think they need the interaction of email or cell phone reminders to keep the process going.”
Did not like it	“It didn't help me, but it didn’t hurt me either.” “What’s needed aren’t pats on the back, a little severity might not hurt.” “To be honest, it didn’t really help me, but thanks anyway.”	“Doesn’t motivate to follow the steps.” “Because when someone is depressed you don’t have the head to read a lot, in fact that’s why I didn’t continue with the modules.” “It’s a lot of content to read, and due to insomnia, my eyes hurt and reading on the computer didn’t help anything.” “Lack of content and motivation.”

#### Design

Open responses regarding the HDep design were also organized into 3 categories, plus one for nonrespondents (Figure 3). Examples of open-ended explanations for the design ratings are presented in Table 9.

The three categories were as follows:

1. Liked the design—more than half of the users liked the design, with no significant difference between HPUs and PUs (HPUs: 35/67, 52%; PUs: 73/124, 58.9%; Figure 3). HPUs found it attractive, user friendly, and colorful; they considered the depression test to be active and they liked how it worked. PUs considered the site neat and well organized, the tools simple, the design motivating, intuitive, and entertaining but perhaps a little too long (Table 9).
2. Had other expectations—fewer participants in each group had other expectations (HPUs: 3/67, 5%; PUs: 4/124, 3.2%). HPUs said they did not receive the expected response or care for their symptoms and had no improvement; PUs believed that the web program, by its nature, reinforced the causes of their depression, not because of problems with its design, but simply because it was a website, meaning that it did not provide contact with a professional, which led to feelings of abandonment (Table 9).
3. Did not like the design—significantly more PUs disliked the design (32/124, 22.8%) than HPUs (9/67, 13%). HPUs noted that HDep was only readable on a computer screen, and it was not encouraging; from their perspective, it should have fewer bright colors and simpler graphics (Table 9).

**Table 9:** Open-ended responses to acceptability of Help for Depression design.

Category	Home page users	Program users
Liked it	“It explains things and invites you to interact with the page.” “It’s user-friendly.” “It’s very colorful, you understand everything” “The evaluation was active, which made it work nicely.” “The design lets you navigate to find the page, I would recommend it.” “It grabs you.”	“Very nicely organized.” “The tools are simple.” “Good design and motivating.” “It’s practical and easy to navigate.” “It’s intuitive and entertaining with graphics, maybe a little long.” “Gets your attention at a glance.”
Had other expectations	“I haven’t gotten a reply.” “I didn’t get attention.” “I haven’t gotten better.”	“I believe that the program, by its nature, reinforced the causes of my depression rather than alleviated them. I mean, it’s not that it’s a bad platform, it’s that it’s a platform.” “Its lack of online specialists on the page was the constant issue I observed with the others in the chat.” “I felt abandoned.”
Did not like it	“Could be more attractive, I don’t know.” “Because if you don’t see it on the computer screen, it’s a bit difficult to read, select and participate. I recommend that you move to a cell phone version in the future.”	“It’s a bit confusing.” “It doesn’t grab your eye, it’s not encouraging.” “It should have softer colors and simpler graphics, something like headspace or calm.” “Not very motivating.”

## Discussion

### Principal Findings

This study explored the satisfaction and acceptability of the updated version of the internet-based preventive intervention, HDep [14] and found them to be consistent with evaluations in a review of digital interventions in LMICs [29]. Two types of users were observed: HPUs, who visit only the home page, make use of the information available there, respond to the CES-D questions, and obtain feedback on their scores and PUs, who also register for a 6-module intervention. The results showed that HPUs and PUs with similar initial levels of depressive symptoms (CES-D>16; 97% and 100%, respectively) showed moderate levels of satisfaction (HPUs: mean 21.90, SD 6.7, range 8-32; PUs: mean 21.10, SD 5.8, range 8-32) and acceptability (HPUs: mean 13.84, SD 3.97, range 5-20; PUs: mean 13.97, SD 3.29, range 5-20). Among HPUs, women expressed higher satisfaction than men, and among PUs, those who were older, employed, not in therapy before, and reported no previous suicide attempts showed higher acceptability.

Of the survey respondents, 52% (35/67) of HPUs and 43.5% (54/124) of PUs reported liking the HDep content; 12% (8/67) and 25% (31/124), respectively, had other expectations, and 12% (8/67) and 16.9% (21/124), respectively, did not like it. With respect to the design, 52% (35/67) of HPUs and 58.9% (73/124) of PUs reported liking it; 5% (3/67) and 3.2% (4/124), respectively, had other expectations, and 13% (9/67) and 25.8% (32/124), respectively, did not like it. Thus, there was no evidence that PUs were more satisfied or indicated a higher degree of acceptability than HPUs, as was hypothesized, based on the fact that the latter received a smaller portion of the intervention. It may be that there is self-selection of users to the dose of the intervention they need. For some, the home page information and feedback about their symptoms seem to be sufficient, whereas others seem to need the entire intervention.

The satisfaction level was above the scale mean, suggesting that users were more satisfied than dissatisfied, to the degree that they “would recommend it to someone in need” or “would use it again if depressed.” Less satisfied participants believed that HDep did not meet their needs. This finding may reflect the high levels of depressive symptoms reported by almost all participants, as well as possible psychopathologies in PUs. Such participants were not the target population of the intervention design, but they were the ones seeking help. Muñoz et al [5] found that this is a common phenomenon, as internet-based interventions intended to be preventive seem to attract individuals who are currently experiencing enough symptoms to screen positive for a major depressive episode; however, only 30% are appropriate for a depression prevention intervention. Other studies have also found a high percentage (up to 90%) of participants in web-based interventions who were highly depressed [30].

Consistent with this notion, higher satisfaction was observed in PUs with less disability owing to depression, no suicidal ideation, and no experience with psychotherapy. According to Rost et al [27], the severity of symptoms and possible comorbidities affect users’ perceptions of acceptability and satisfaction. Self-guided interventions have been observed to be more efficacious for users with less psychotherapeutic experience [31].

Findings from the World Mental Health Survey show that in Mexico, 58.3% of people with a diagnosis of major depressive disorder who felt they needed treatment, only 6.4% received treatment that was minimally adequate [3]. This situation may explain why many people with probable mental health disorders seek help in web-based interventions. One-third of HDep users said they had, at some point in the past, sought either psychological or psychiatric mental health treatment; a similar web-based intervention for depression reported by Christensen et al [32] also found that 64% of participants had previously sought professional help. Our findings show that users with previous experience in therapy were least satisfied and gave HDep lower acceptability ratings. Participants with this experience could be advised early on about what they could realistically expect from HDep.

The general acceptability ratings of HDep were above the mean, suggesting that users considered it acceptable but with room for improvement. Some of the features they valued were its user-friendliness and usefulness in managing their depression, but it did not fully meet their expectations. Regarding the content, more than half of the respondents described it as good, meaningful, and helpful in making them feel better, friendly, systematic, well-paced, and encouraging reflection. More than half of the respondents liked the design, describing it as easy to understand, well organized, attractive, user friendly, colorful, intuitive, and entertaining.

The test for depressive symptoms and feedback regarding results had the highest acceptability ratings, whereas forums and chats had the lowest ratings (Table 6). Some studies have found a high degree of satisfaction with mood-monitoring rating tools in web-based treatment for depression [33]. Questionnaires and psychometric tests sometimes give participants a feeling of personalized treatment [34]. The acceptability of HDep and similar interventions may therefore be improved by increasing the use of these tools.

It is noteworthy that forums received low ratings, although it has previously been observed that forums allow for meaningful social exchange of experiences [15]. One possible explanation may lie in the observation that although a large proportion of participants used forums (60.9%), far fewer posted comments (16.3%) [15]. The lack of participation of a professional in forums and chats is also a possible reason for the low ratings.

Three major areas were found to be lacking in the design and content of HDep. These were described as (1) not being sufficiently persuasive, dynamic, motivating, or appealing; (2) not meeting users’ expectations; and (3) being cold, being *just a platform*, and lacking professionals to support, advise, and track people’s progress. Other studies have also found that participants in automated programs are concerned about their being too impersonal [35]. Therapeutic persuasiveness, the incorporation of persuasive principles of design, and behavior change have been described as the most robust predictors of adherence [36]. Not meeting users’ expectations may be particularly important for depressed users, because as Bernard et al [37] reported, these users become particularly upset when they find unexpected, irrelevant, or inappropriate content. Despite the importance of design in developing successful web-based interventions, Neilsen and Wilson [38] concluded in a literature review that design elements and human–computer interaction remain poorly understood and that “internet-based e-mental health interventions are routinely implemented without sufficiently describing the relevant human–computer interaction design features applied.” In this respect, HDep would benefit from incorporating a user-centered design approach to improve the layout of its content [29].

The content deficiency perceived by HDep users was the need for professional help or specialized guidance. This issue has often been reported in the literature on unguided interventions [8,15], and it also turned out to be important for Mexican users in our survey. One of the most attractive aspects of web-based interventions for mental health problems is their reduced cost, including personalized professional help, which would be a significant burden. This is very much the case for HDep, which is supported by a public health institution as a translational research project. Features to compensate for the lack of professional support could include automated dialogue components, such as automated SMS text messages or email messages or gaming features [38].

### Conclusions

Overall, HDep showed moderate levels of satisfaction and acceptability and high levels for more than half of its participants, despite a level of depressive symptoms high enough to suggest a major depressive episode. HPUs and PUs rated satisfaction and acceptability similarly, contrary to our hypothesis that the latter, having received a larger portion of the intervention, would be more satisfied. Respondents’ ratings on satisfaction and acceptability may be related to their ability to choose how much of the program they want to do: whether they just want an overview from the home page or want to work on the intervention modules. They considered the content culturally sensitive, reflecting their everyday experiences; they found the design to be friendly, with tools that were well organized, simple, and motivating. The main limitations were the lack of contact with a professional, and in some cases, content that did not motivate and was not encouraging. Users who responded to the survey provided abundant suggestions on feasible ways to alleviate some of the deficiencies. Some of these deficiencies coincide with those observed in other web-based interventions for depression [8,27,35,38].

This study has the strength of being based in a real-world setting, not in a confined research environment, so that it reflects more closely what real participants do. However, this feature also makes it more difficult to implement strict methodological control. HDep is the first web-based self-help intervention for depression in Latin America and has been in operation for 11 years. It is a promising and cost-effective tool that can contribute to reducing the treatment gap for depression in Mexico [3]. It has the potential to provide mental health literacy to a large group of users and can be integrated into a preventive stepped-care approach [39]. Being a qualitative–quantitative study from a non–English-speaking LMIC, this study adds to existing research on acceptance and satisfaction with cognitive behavioral therapy–based programs for depression in high-income countries. There appear to be no studies directly comparing users’ performance in web-based interventions to assess differences among countries with different income levels. These could include cultural differences: English-speaking users of web-based interventions tend to emphasize the benefits of introspection and self-awareness, which is congruent with *the dominant individualized focus of Euro-American cultural orientation compared to a more collectivist and relational Latin American cultural orientation* [40].

Although there are many effective internet-based interventions for depression, there are far fewer with open access for general use [41]. Web-based interventions have great potential in Mexico, where it is estimated that 80.6 million people use the internet [42], 58% of whom are interested in health content [43]. The findings of this study are important for the creation and adaptation of web-based interventions in an LMIC, such as Mexico, where access to treatment is a major concern [2] and web-based prevention and treatment programs can help to deliver evidence-based alternatives. HDep is promising, but it is necessary to further document the pitfalls, strengths, and challenges of this type of intervention in this particular context.

### Limitations

A major limitation of this study was its response rate of 1.45% (191/13,207). This response implied a CI of 95% and a margin of error of 7%. Low response rates are an important concern that affects the validity of web surveys [44]. In a meta-analytic study, Daikeler et al [21] found that web surveys yielded a response rate that was 12% points lower than other survey modes and concluded that the difference is not the result of a particular study design. They also found evidence that low response rates do not necessarily indicate a large nonresponse error.

Participants who did not respond may have been less satisfied and less positive than those who did [35]. The levels of depressive symptoms and other mental health problems of the people surveyed may explain the low response rate; people with emotional stability are more likely to complete a survey [44]. It is also likely that many of the people contacted were no longer using the intervention, as the survey was sent to those registered in the previous year. There was just 1 reminder email; additional reminders may have increased the response rates [21]. Some of those contacted may have visited HDep just once. It may also be that the incentive (receiving a list of positive thoughts to practice) was not found attractive [44]. There was also no confirmation that users received the survey; email questionnaires are often treated as spam or blocked [21,44].

To estimate other possible sources of bias, the characteristics of the study sample were compared with those of a previous study on HDep [15], which obtained data directly from registered users. No differences >10% were found between the 2 samples in terms of sex, depressive symptoms, disability, suicide attempts, previous psychological or psychiatric treatment, alcohol or drug use, or drug treatment. The proportion of users aged >30 years was >12.5% in the current sample. The proportion of HPUs and PUs in this study was similar to that in a previous study [15]: HPUs made up 35.1% (67/191) of respondents in this study and 38.32% (10,760/28,078) in the previous study; PUs made up 64.9% (124/191) and 61.69% (17,318/28,078), respectively. These figures suggest that the low response rate did not skew the sample with respect to these variables, but there could still be other biases, such as a self-selection bias of eagerness to respond.

## Abbreviations

CES-D: Center for Epidemiological Studies Scale-Depression
CSQ-8: Client Satisfaction Questionnaire-8
HDep: Help for Depression (in Spanish, ADep: Ayuda Para Depresión)
HPU: home page user
LMIC: low- and middle-income country
OR: odds ratio
PU: program user
